# The lower bounds for the rank of matrices and some sufficient conditions for nonsingular matrices

**DOI:** 10.1186/s13660-017-1446-4

**Published:** 2017-07-20

**Authors:** Dafei Wang, Xumei Zhang

**Affiliations:** School of Economics and Business Administration, Chongqing University, Chongqing, 400044 P.R. China

**Keywords:** 47A63, nonsingular matrices, lower bounds, rank, sufficient conditions

## Abstract

The paper mainly discusses the lower bounds for the rank of matrices and sufficient conditions for nonsingular matrices. We first present a new estimation for $\sum_{i=1}^{n} \vert \lambda_{i} \vert ^{2}$ ($\lambda_{i}$ is an eigenvalue of a matrix) by using the partitioned matrices. By using this estimation and inequality theory, the new and more accurate estimations for the lower bounds for the rank are deduced. Furthermore, based on the estimation for the rank, some sufficient conditions for nonsingular matrices are obtained.

## Introduction

Let $M_{n}(C)$ be the set of $n\times n$ complex matrix. Let $A=(a_{ij})_{n\times n}\in M_{n}(C)$. Denote by $A^{\ast}$, $\Vert A \Vert _{F}$, $\mathrm{r}(A)$ and tr*A* the conjugate transpose, Frobenius norm, rank and trace of *A*, respectively. Let $[A,A^{\ast}]=AA^{\ast}-A^{\ast}A $.

The lower bounds for the rank of matrices play an important role in diagnosing nonsingular matrices. A well-known inequality for $\mathrm {r}(A)$ given by Ky Fan and Hoffman [1], is as follows:

1.1 $$ \mathrm{r}(A)\geq\sum_{i=1}^{n} \frac{ \vert a_{ii} \vert }{\sum_{j=1}^{n} \vert a_{ij} \vert }. $$

Huang and You [2] improved the Ky Fan and Hoffman’s inequality, as follows:

1.2 $$ \mathrm{r}(A)\geq\frac{ \vert \operatorname{tr}A \vert ^{2}}{\sum_{i=1}^{n} (\sum_{j=1}^{n} \vert a_{ij} \vert ^{2} )^{\frac{1}{2}} (\sum_{j=1}^{n} \vert a_{ji} \vert ^{2} )^{\frac{1}{2}}}. $$

Let *M* be an $n\times n$ complex matrix and partitioned as

$$M=\left [ \begin{matrix}A_{k\times k} & B_{k\times(n-k)}\\ C_{(n-k)\times k} & D_{(n-k)\times(n-k)} \end{matrix} \right ]\quad (1\leq k\leq n-1), $$

where $A_{k\times k}$ is a $k\times k$ principal submatrix of *M*. In [3], the inequality of lower bound of rank was shown that

1.3 $$ \mathrm{r}(M)\geq\frac{ \vert \operatorname{tr}M \vert ^{2}}{ \Vert M \Vert ^{2}_{F}- ( \Vert B_{k\times (n-k)} \Vert _{F}- \Vert C_{(n-k)\times k } \Vert _{F} )^{2}}. $$

In this paper, some new estimations about the lower bounds for the rank are deduced, which improve the above estimations. In order to facilitate the expression, we define the following forms of representation throughout this paper:

$$\begin{aligned} &\varphi_{M}(k)= \Vert M \Vert ^{2}_{F}- \bigl( \Vert B_{k\times(n-k)} \Vert _{F}- \Vert C_{(n-k)\times k } \Vert _{F} \bigr)^{2}-\frac{ \vert \operatorname{tr}M \vert ^{2}}{n}, \\ &\varphi_{M}(k,x)= \Vert M \Vert ^{2}_{F}- \bigl[\bigl(1-x^{2}\bigr) \Vert B_{k\times(n-k)} \Vert _{F}^{2}+\bigl(1-x^{-2}\bigr) \Vert C_{(n-k)\times k } \Vert ^{2}_{F} \bigr]-\frac{ \vert \operatorname{tr}M \vert ^{2} }{n}, \\ &M_{k}(x)=\left [ \begin{matrix}A_{k\times k} & xB_{k\times(n-k)}\\ x^{-1}C_{(n-k)\times k} & D_{(n-k)\times(n-k)} \end{matrix} \right ],\qquad M_{k}=\left [ \begin{matrix}A_{k\times k} & \sqrt{\mu}B_{k\times(n-k)}\\ \sqrt{1/\mu }C_{(n-k)\times k} & D_{(n-k)\times(n-k)} \end{matrix} \right ], \end{aligned}$$

where *x* is a non-zero real number, $\mu=\frac{ \Vert C_{(n-k)\times k} \Vert _{F}}{ \Vert B_{k\times(n-k) } \Vert _{F}}\neq0$.

## Estimations for the lower bounds for the rank

In this section, some new estimations about lower bounds for the rank are obtained. We first give the following lemma.

### Lemma 2.1

*Let* $M \in M_{n}(C)$ *be an* $n\times n$ *complex matrix with eigenvalues* $\lambda_{i}$ ($i=1,2,\ldots,n$). *Then*

$$\sum_{i=1}^{n} \vert \lambda_{i} \vert ^{2}\leq \biggl[\bigl(\varphi _{M}(k,x)\bigr)^{2}-\frac{1}{2} \bigl\Vert \bigl[M_{k}(x),M_{k}(x)^{\ast}\bigr] \bigr\Vert ^{2}_{F} \biggr]^{\frac{1}{2}}+\frac{ \vert \operatorname {tr}M \vert ^{2}}{n}. $$

### Proof

Let $R=M_{k}(x)-\frac{\operatorname{tr}M}{n}I$, where *I* is an $n\times n$ unit matrix. We note that $M_{k}(x)$ is similar to *M*, then $M_{k}(x)$ has the same eigenvalues with *M*, *i.e.*, $\lambda_{i}$ is the eigenvalues of $M_{k}(x)$. So, we can deduce that $\lambda_{i}-\frac{\operatorname{tr}M}{n}$ ($i=1,2,\ldots,n$) are eigenvalues of *R*. According to the Kress theorem in [4], we have

$$\sum_{i=1}^{n} \biggl\vert \lambda_{i}-\frac{\operatorname{tr}M}{n} \biggr\vert ^{2}\leq \biggl( \Vert R \Vert ^{4}_{F}-\frac{1}{2} \bigl\Vert \bigl[R,R^{\ast}\bigr] \bigr\Vert ^{2}_{F} \biggr)^{\frac{1}{2}}. $$

We note the following equalities:

$$\begin{gathered} \sum_{i=1}^{n} \biggl\vert \lambda_{i}-\frac{\operatorname{tr}M}{n} \biggr\vert ^{2}=\sum _{i=1}^{n} \vert \lambda_{i} \vert ^{2}-\frac { \vert \operatorname{tr}M \vert ^{2}}{n}, \\ \bigl[R,R^{\ast}\bigr]= \biggl[M_{k}(x)-\frac{\operatorname{tr}M}{n}I,M_{k}(x)^{\ast }- \frac{\overline{\operatorname{tr}M}}{n}I \biggr]=\bigl[M_{k}(x),M_{k}(x)^{\ast} \bigr], \\ \Vert R \Vert ^{2}_{F}=\operatorname{tr} \biggl[ \biggl(M_{k}(x)-\frac{\operatorname{tr}M}{n}I \biggr) \biggl(M_{k}(x)- \frac {\operatorname{tr}M}{n}I \biggr)^{\ast} \biggr]= \bigl\Vert M_{k}(x) \bigr\Vert ^{2}_{F}- \frac{ \vert \operatorname{tr}M \vert ^{2}}{n}=\varphi_{M}(k,x). \end{gathered} $$

Combining the above conclusions, we can directly deduce Lemma 2.1. □

### Theorem 2.1

*Let* $M \in M_{n}(C)$ *be an* $n\times n$ *complex matrix*, *then*

2.1 $$ \mathrm{r}(M)\geq\frac{ \vert \operatorname{tr}M \vert ^{2}}{ [(\varphi_{M}(k,x))^{2}-\frac{1}{2} \Vert [M_{k}(x),M_{k}(x)^{\ast}] \Vert ^{2}_{F} ]^{\frac {1}{2}}+\frac{ \vert \operatorname{tr}M \vert ^{2}}{n}}. $$

### Proof

By the Schur theorem, there is a unitary matrix $U\in M_{n}(C)$ such that $UMU^{\ast}$ is upper triangular, *i.e.*,

$$UMU^{\ast}=\left [ \begin{matrix} \lambda_{1} & & & * \\ &\lambda_{2}& \\ & &\ddots& \\ 0& & &\lambda_{n} \end{matrix} \right ], $$

where $\lambda_{1},\lambda_{2},\ldots,\lambda_{n}$ are eigenvalues of *M*. Without loss of generality, we suppose $\lambda_{1},\lambda _{2},\ldots, \lambda_{p}$ are all non-zero eigenvalues of *M*, then we can get

$$\vert \operatorname{tr}M \vert ^{2}= \bigl\vert \operatorname {tr}\bigl(UMU^{\ast}\bigr) \bigr\vert ^{2}= \Biggl\vert \sum _{i=1}^{n}\lambda _{i} \Biggr\vert ^{2}= \Biggl\vert \sum_{i=1}^{p} \lambda_{i} \Biggr\vert ^{2}\leq p\sum _{i=1}^{p} \vert \lambda_{i} \vert ^{2}\leq \mathrm{r}(M)\sum_{i=1}^{p} \vert \lambda_{i} \vert ^{2}. $$

Applying Lemma 2.1, we have

$$\vert \operatorname{tr}M \vert ^{2}\leq\mathrm{r}(M) \biggl[ \biggl(\bigl(\varphi_{M}(k,x)\bigr)^{2}-\frac{1}{2} \bigl\Vert \bigl[M_{k}(x),M_{k}(x)^{\ast}\bigr] \bigr\Vert ^{2}_{F} \biggr)^{\frac {1}{2}}+ \frac{ \vert \operatorname{tr}M \vert ^{2}}{n} \biggr]. $$

Thus, the proof is completed. □

Now let us consider some special cases of this theorem, the cases $x=1$ and $x=\sqrt{\frac{ \Vert C_{(n-k)\times k} \Vert _{F}}{ \Vert B_{k\times(n- k)} \Vert _{F}}}$, and we have the following corollary.

### Corollary 2.1

*Let* $M \in M_{n}(C)$ *be an* $n\times n$ *complex matrix*, *then*

2.2 $$\begin{aligned} &(1)\quad \mathrm{r}(M)\geq\frac{ \vert \operatorname{tr}M \vert ^{2}}{ [ ( \Vert M \Vert ^{2}_{F}-\frac{ \vert \operatorname{tr}M \vert ^{2}}{n} )^{2}-\frac{1}{2} \Vert [M,M^{*}] \Vert _{F}^{2} ]^{\frac{1}{2}}+\frac{ \vert \operatorname{tr}M \vert ^{2}}{n}}, \end{aligned}$$

2.3 $$\begin{aligned} &(2)\quad \mathrm{r}(M)\geq\frac{ \vert \operatorname{tr}M \vert ^{2}}{ [(\varphi_{M}(k))^{2}-\frac{1}{2} \Vert [M_{k},M^{*}_{k}] \Vert _{F}^{2} ]^{\frac{1}{2}}+\frac{ \vert \operatorname{tr}M \vert ^{2}}{n}}. \end{aligned}$$

### Theorem 2.2

*Let* $M \in M_{n}(C)$ *be an* $n\times n$ *complex matrix with all non*-*zero eigenvalues* $\lambda_{1},\lambda_{2},\ldots,\lambda_{p}$, $p\geq2$, *then*

2.4 $$\begin{aligned} &\mathrm{r}(M)\geq\frac{ \vert \operatorname{tr}M \vert ^{2}+\frac{p}{2}\max_{i,j=1,2,\ldots,p} \vert \lambda_{i}-\lambda _{j} \vert ^{2}}{ [(\varphi_{M}(k,x))^{2}-\frac{1}{2} \Vert [M_{k}(x),M_{k}(x)^{*}] \Vert ^{2}_{F} ]^{\frac{1}{2}}+\frac { \vert \operatorname{tr}M \vert ^{2}}{n}}, \end{aligned}$$

2.5 $$\begin{aligned} &\mathrm{r}(M)\geq\frac{ \vert \operatorname{tr}M \vert ^{2}}{ [(\varphi_{M}(k,x))^{2}-\frac{1}{2} \Vert [M_{k}(x),M_{k}(x)^{*}] \Vert ^{2}_{F} ]^{\frac{1}{2}}+\frac { \vert \operatorname{tr}M \vert ^{2}}{n}-\frac{1}{2}\max_{i,j=1,2,\ldots,p} \vert \lambda_{i}-\lambda_{j} \vert ^{2}}. \end{aligned}$$

### Proof

Without loss of generality, let $\max_{i,j=1,2,\ldots,p} \vert \lambda_{i}-\lambda_{j} \vert = \vert \lambda_{1}-\lambda _{p} \vert $. By Lagrange’s identity, we have

2.6 $$\begin{aligned} p\sum_{i=1}^{p} \vert \lambda_{i} \vert ^{2}- \Biggl\vert \sum _{i=1}^{p}\lambda_{i} \Biggr\vert ^{2} =&\sum_{1\leq i< j\leq p} \vert \lambda_{i}-\lambda_{j} \vert ^{2} \\ =& \vert \lambda_{1}-\lambda_{p} \vert ^{2}+\sum_{j=2}^{p-1} \vert \lambda_{j}-\lambda_{p} \vert ^{2}+ \sum_{j=2}^{p-1}\vert \lambda_{1} -\lambda_{j}\vert^{2}+\sum _{2\leq i< j\leq p-1} \vert \lambda _{i}-\lambda_{j} \vert ^{2} \\ =& \vert \lambda_{1}-\lambda_{p} \vert ^{2}+\sum_{j=2}^{p-1}\bigl( \vert \lambda_{1}-\lambda_{j} \vert ^{2}+ \vert \lambda_{j}-\lambda_{p} \vert ^{2}\bigr) +\sum _{2\leq i< j\leq p-1} \vert \lambda_{i}- \lambda_{j} \vert ^{2} \\ \geq& \vert \lambda_{1}-\lambda_{p} \vert ^{2}+\sum_{j=2}^{p-1} \frac{ \vert \lambda_{1}-\lambda_{p} \vert ^{2}}{2}+\sum_{2\leq i< j\leq p-1} \vert \lambda_{i}-\lambda _{j} \vert ^{2} \\ \geq& \vert \lambda_{1}-\lambda_{p} \vert ^{2}+\frac {p-2}{2} \vert \lambda_{1}- \lambda_{p} \vert ^{2}=\frac {p}{2} \vert \lambda_{1}-\lambda_{p} \vert ^{2} . \end{aligned}$$

According to Lemma 2.1, we get

$$p \biggl[\bigl(\varphi_{M}(k,x)\bigr)^{2}- \frac{1}{2} \bigl\Vert \bigl[M_{k}(x),M_{k}(x)^{*} \bigr] \bigr\Vert _{F}^{2})^{\frac{1}{2}}+ \frac{ \vert \operatorname{tr}M \vert ^{2}}{n} \biggr]- \vert \operatorname{tr}M \vert ^{2}\geq \frac{p}{2} \vert \lambda _{1}-\lambda_{p} \vert ^{2}, $$

*i.e.*,

$$p\geq\frac{ \vert \operatorname{tr}M \vert ^{2}}{ [(\varphi _{M}(k,x))^{2}-\frac{1}{2} \Vert [M_{k}(x),M_{k}(x)^{*}] \Vert _{F}^{2} ]^{\frac{1}{2}}+\frac{ \vert \operatorname{tr}M \vert ^{2}}{n}-\frac{1}{2} \vert \lambda_{1}-\lambda_{p} \vert ^{2}}. $$

We know that $\mathrm{r}(M)\geq p$, therefore the conclusion (2.5) is true.

Applying Lemma 2.1 and (2.6), we can get

$$\mathrm{r}(M)\geq\frac{ \vert \operatorname{tr}M \vert ^{2}+\frac{p}{2} \vert \lambda_{1}-\lambda_{p} \vert ^{2}}{ [(\varphi_{M}(k,x))^{2}-\frac{1}{2} \Vert [M_{k}(x),M_{k}(x)^{*}] \Vert _{F}^{2} ]^{\frac{1}{2}}+\frac { \vert \operatorname{tr}M \vert ^{2}}{n}}. $$

The proof is completed. □

### Corollary 2.2

*Let* $M \in M_{n}(C)$ *be an* $n\times n$ *complex matrix with all non*-*zero eigenvalues* $\lambda_{1},\lambda_{2},\ldots,\lambda_{p}$, $p\geq2$; *then*

$$\begin{aligned} &(1)\quad \mathrm{r}(M)\geq\frac{ \vert \operatorname{tr}M \vert ^{2}}{ [ ( \Vert M \Vert ^{2}_{F}-\frac{ \vert \operatorname{tr}M \vert ^{2}}{n} )^{2}-\frac{1}{2} \Vert [M,M^{*}] \Vert ^{2}_{F} ]^{\frac{1}{2}}+\frac{ \vert \operatorname{tr}M \vert ^{2}}{n}-\frac{1}{2}\max_{i,j=1,2,\ldots,p} \vert \lambda_{i}-\lambda_{j} \vert ^{2}}, \\ &(2)\quad \mathrm{r}(M)\geq\frac{ \vert \operatorname{tr}M \vert ^{2}+\frac{p}{2}\max_{i,j=1,2,\ldots,p} \vert \lambda _{i}-\lambda_{j} \vert ^{2}}{ [ ( \Vert M \Vert ^{2}_{F}-\frac{ \vert \operatorname{tr}M \vert ^{2}}{n} )^{2}-\frac{1}{2} \Vert [M,M^{*}] \Vert ^{2}_{F} ]^{\frac {1}{2}}+\frac{ \vert \operatorname{tr}M \vert ^{2}}{n}}, \\ &(3)\quad \mathrm{r}(M)\geq\frac{ \vert \operatorname{tr}M \vert ^{2}}{ [ (\varphi_{M}(k)-\frac{ \vert \operatorname {tr}M \vert ^{2}}{n} )^{2}-\frac{1}{2} \Vert [M_{k},M_{k}^{*}] \Vert ^{2}_{F} ]^{\frac{1}{2}}+\frac{ \vert \operatorname{tr}M \vert ^{2}}{n}-\frac{1}{2}\max_{i,j=1,2,\ldots,p} \vert \lambda_{i}-\lambda_{j} \vert ^{2}}, \\ &(4)\quad \mathrm{r}(M)\geq\frac{ \vert \operatorname{tr}M \vert ^{2}+\frac{p}{2}\max_{i,j=1,2,\ldots,p} \vert \lambda _{i}-\lambda_{j} \vert ^{2}}{ [ (\varphi_{M}(k)-\frac{ \vert \operatorname{tr}M \vert ^{2}}{n} )^{2}-\frac{1}{2} \Vert [M_{k},M_{k}^{*}] \Vert ^{2}_{F} ]^{\frac{1}{2}}+\frac { \vert \operatorname{tr}M \vert ^{2}}{n}}. \end{aligned}$$

### Theorem 2.3

*Let* $M \in M_{n}(C)$ *be an* $n\times n$ *complex matrix with eigenvalues* $\lambda_{i}=a_{i}+b_{i}\sqrt{-1}$ ($i=1,2,\ldots,n$), *where* $a_{i}$, $b_{i}$ *denote the real parts and imaginary parts of* $\lambda_{i}$, *respectively* ($a_{i}=\operatorname{Re}(\lambda_{i})$, $b_{i}=\operatorname {Im}(\lambda_{i})$). *Then*

2.7 $$\begin{aligned} \mathrm{r}(M)&\geq\frac{ \vert \operatorname{Re}(\operatorname {tr}M) \vert ^{2}}{ \Vert \frac{M+M^{*}}{2} \Vert ^{2}_{F}-\frac{1}{2} \Vert M \Vert ^{2}_{F}+\frac{1}{2} [ ((\varphi_{M}(k,x))^{2} -\frac{1}{2} \Vert [M_{k}(x),M_{k}(x)^{*}] \Vert ^{2}_{F} )^{\frac{1}{2}}+\frac{ \vert \operatorname{tr}M \vert ^{2}}{n} ]}, \end{aligned}$$

2.8 $$\begin{aligned} \mathrm{r}(M)&\geq\frac{ \vert \operatorname{Im}(\operatorname {tr}M) \vert ^{2}}{ \Vert \frac{M-M^{*}}{2} \Vert ^{2}_{F}-\frac{1}{2} \Vert M \Vert ^{2}_{F} +\frac{1}{2} [ ((\varphi_{M}(k,x))^{2}-\frac{1}{2} \Vert [M_{k}(x),M_{k}(x)^{*}] \Vert ^{2}_{F} )^{\frac{1}{2}}+\frac { \vert \operatorname{tr}M \vert ^{2}}{n} ]}. \end{aligned}$$

### Proof

By the Schur theorem, there is a unitary matrix *U* such that $UMU^{*}$ is an upper triangular matrix, *i.e.*,

$$UMU^{*}=\left [ \begin{matrix} \lambda_{1} & d_{12}&d_{13}& \cdots&d_{1n} \\ 0&\lambda_{2}&d_{23}& \cdots&d_{2n} \\ \vdots& \vdots& \vdots& & \vdots\\ 0&0&0&\cdots&\lambda_{n} \end{matrix} \right ], $$

where $\lambda_{1},\lambda_{2},\ldots,\lambda_{n}$ are eigenvalues of *M*. Since the Frobenius norm is unitarily invariant norm, we can deduce that

2.9 $$ \bigl\Vert UMU^{*} \bigr\Vert ^{2}_{F}= \Vert M \Vert ^{2}_{F}=\sum _{i=1}^{n} \vert \lambda_{i} \vert ^{2}+\sum_{1\leq i< j\leq n} \vert d_{ij} \vert ^{2}. $$

Furthermore,

$$U \biggl(\frac{M+M^{*}}{2} \biggr)U^{*}=\left [ \begin{matrix} a_{1} & \frac{1}{2}d_{12}&\frac{1}{2}d_{13}& \cdots&\frac{1}{2}d_{1n} \\ \frac{1}{2}\overline{d_{12}}&a_{2}& \frac{1}{2}d_{23}& \cdots&\frac {1}{2}d_{2n} \\ \vdots& \vdots& \vdots& & \vdots\\ \frac{1}{2}\overline{d_{1n}}&\frac{1}{2}\overline{d_{2n}}& \frac {1}{2}\overline{d_{3n}}& \cdots&a_{n} \end{matrix} \right ], $$

where $a_{i}=\operatorname{Re}(\lambda_{i})$ ($i=1,2,\ldots,n$), and by the unitarily invariant norm

2.10 $$ \biggl\Vert U \biggl(\frac{M+M^{*}}{2} \biggr)U^{*} \biggr\Vert ^{2}_{F}= \biggl\Vert \frac{M+M^{*}}{2} \biggr\Vert ^{2}_{F}=\sum_{i=1}^{n}a_{i}^{2}+ \frac{1}{2} \sum_{1\leq i< j\leq n}^{n} \vert d_{ij} \vert ^{2}. $$

Similarly we have available

2.11 $$ \biggl\Vert \frac{M-M^{*}}{2\sqrt{-1}} \biggr\Vert ^{2}_{F}=\sum_{i=1}^{n}b_{i}^{2}+ \frac{1}{2} \sum_{1\leq i< j\leq n} \vert d_{ij} \vert ^{2}, $$

where $b_{i}=\operatorname{Im}(\lambda_{i})$ ($i=1,2,\ldots,n$). Combining (2.9) and (2.10), we can deduce that

$$\sum_{i=1}^{n}a_{i}^{2}= \biggl\Vert \frac{M+M^{*}}{2} \biggr\Vert ^{2}_{F}- \frac{1}{2} \Biggl( \Vert M \Vert ^{2}_{F}-\sum _{i=1}^{n} \vert \lambda_{i} \vert ^{2} \Biggr). $$

Applying Lemma 2.1, we get

2.12 $$ \begin{aligned}[b]\sum_{i=1}^{n}a_{i}^{2} &\leq \biggl\Vert \frac{M+M^{*}}{2} \biggr\Vert ^{2}_{F}- \frac{1}{2} \Vert M \Vert ^{2}_{F}\\ &\quad {}+ \frac{1}{2} \biggl[ \biggl(\bigl(\varphi_{M}(k,x) \bigr)^{2} -\frac{1}{2} \bigl\Vert \bigl[M_{k}(x),M_{k}(x)^{*} \bigr] \bigr\Vert ^{2}_{F} \biggr)^{\frac{1}{2}}+ \frac{ \vert \operatorname{tr}M \vert ^{2}}{n} \biggr]. \end{aligned} $$

Similarly, according to (2.9), (2.11) and Lemma 2.1, we can deduce that

2.13 $$ \begin{aligned}[b] \sum_{i=1}^{n}b_{i}^{2} &\leq \biggl\Vert \frac{M-M^{*}}{2\sqrt{-1}} \biggr\Vert ^{2}_{F}- \frac{1}{2} \Vert M \Vert ^{2}_{F}\\ &\quad {} + \frac{1}{2} \biggl[ \biggl(\bigl(\varphi_{M}(k,x) \bigr)^{2}-\frac{1}{2} \bigl\Vert \bigl[M_{k}(x),M_{k}(x)^{*} \bigr] \bigr\Vert ^{2}_{F} \biggr)^{\frac{1}{2}}+ \frac { \vert \operatorname{tr}M \vert ^{2}}{n} \biggr]. \end{aligned} $$

Without loss of generality, let $\lambda_{1},\lambda_{2},\ldots,\lambda {_{t}}$ be all non-zero eigenvalues of *M*, so there are no more *t* non-zero real parts $a_{i_{1}},a_{i_{2}},\ldots,a_{i_{k}}$ ($k\leq t$) in $a_{1},a_{2},\ldots,a_{n}$. Thus, we can deduce the following conclusion:

2.14 $$ \mathrm{r}(M)\geq t\geq k\geq\frac{ (\sum_{h=1}^{k}a_{i_{h}} )^{2}}{\sum_{h=1}^{k}a_{i_{h}}^{2}}= \frac{ \vert \operatorname {Re}(\operatorname{tr}M) \vert ^{2}}{\sum_{i=1}^{n}a_{i}^{2}}. $$

Similarly we have available

2.15 $$ \mathrm{r}(M)\geq\frac{ \vert \operatorname{Im}(\operatorname {tr}M) \vert ^{2}}{\sum_{i=1}^{n}b_{i}^{2}}. $$

By (2.12) and (2.14), we can directly get the conclusion (2.7). In the same way, by (2.13) and (2.15), we can also directly get the conclusion (2.8). The proof is completed. □

### Corollary 2.3

*Let* $M \in M_{n}(C)$ *be an* $n\times n$ *complex matrix*. *Then*

2.16 $$\begin{aligned} &\mathrm{r}(M)\geq\frac{ \vert \operatorname{Re}(\operatorname {tr}M) \vert ^{2}}{ \Vert \frac{M+M^{*}}{2} \Vert ^{2}_{F}-\frac{1}{2} \Vert M \Vert ^{2}_{F} +\frac{1}{2} [ ( ( \Vert M \Vert ^{2}_{F}-\frac { \vert \operatorname{tr}M \vert ^{2}}{n} )^{2}-\frac {1}{2} \Vert [M,M^{*}] \Vert ^{2}_{F} )^{\frac {1}{2}}+\frac{ \vert \operatorname{tr}M \vert ^{2}}{n} ]}, \end{aligned}$$

2.17 $$\begin{aligned} &\mathrm{r}(M)\geq\frac{ \vert \operatorname{Im}(\operatorname {tr}M) \vert ^{2}}{ \Vert \frac{M-M^{*}}{2\sqrt{-1}} \Vert ^{2}_{F}-\frac{1}{2} \Vert M \Vert ^{2}_{F}+\frac {1}{2} [ ( ( \Vert M \Vert ^{2}_{F} -\frac{ \vert \operatorname{tr}M \vert ^{2}}{n} )^{2}-\frac{1}{2} \Vert [M,M^{*}] \Vert ^{2}_{F} )^{\frac {1}{2}}+\frac{ \vert \operatorname{tr}M \vert ^{2}}{n} ]}, \end{aligned}$$

2.18 $$\begin{aligned} &\mathrm{r}(M)\geq\frac{ \vert \operatorname{Re}(\operatorname {tr}M) \vert ^{2}}{ \Vert \frac{M+M^{*}}{2} \Vert ^{2}_{F}-\frac{1}{2} \Vert M \Vert ^{2}_{F}+\frac{1}{2} [ ((\varphi_{M}(k))^{2} -\frac{1}{2} \Vert [M_{k},M^{*}_{k}] \Vert ^{2}_{F} )^{\frac{1}{2}}+\frac{ \vert \operatorname{tr}M \vert ^{2}}{n} ]}, \end{aligned}$$

2.19 $$\begin{aligned} &\mathrm{r}(M)\geq\frac{ \vert \operatorname{Im}(\operatorname {tr}M) \vert ^{2}}{ \Vert \frac{M-M^{*}}{2\sqrt{-1}} \Vert ^{2}_{F}-\frac{1}{2} \Vert M \Vert ^{2}_{F}+\frac {1}{2} [ ((\varphi_{M}(k))^{2} -\frac{1}{2} \Vert [M_{k},M^{*}_{k}] \Vert ^{2}_{F} )^{\frac{1}{2}}+\frac{ \vert \operatorname{tr}M \vert ^{2}}{n} ]}. \end{aligned}$$

## Some sufficient conditions for nonsingular matrices

In this section, based on the conclusions of Section 2, we directly obtain some simple sufficient conditions for nonsingular matrices.

According to Theorem 2.1, we can directly get the following.

### Theorem 3.1

*Let* $M \in M_{n}(C)$; *if* *M* *satisfies the following condition*:

$$\vert \operatorname{tr}M \vert ^{2}>(n-1) \biggl[ \biggl(\bigl( \varphi _{M}(k,x)\bigr)^{2}-\frac{1}{2} \bigl\Vert \bigl[M_{k}(x),M_{k}(x)^{*}\bigr] \bigr\Vert ^{2}_{F} \biggr)^{\frac{1}{2}} +\frac{ \vert \operatorname{tr}M \vert ^{2}}{n} \biggr], $$

*then* *M* *is nonsingular matrix*.

According to Corollary 2.1, we have the following.

### Corollary 3.1

*Let* $M \in M_{n}(C)$; *if* *M* *satisfies one of the following conditions*, *then* *M* *is nonsingular matrix*:

$$\begin{aligned} &(1)\quad \vert \operatorname{tr}M \vert ^{2}> (n-1) \biggl[ \biggl( \biggl( \Vert M \Vert ^{2}_{F}- \frac{\vert\operatorname{tr}M\vert^{2}}{n} \biggr)^{2} -\frac{1}{2} \bigl\Vert \bigl[M,M^{*}\bigr] \bigr\Vert ^{2}_{F} \biggr)^{\frac {1}{2}}+\frac{ \vert \operatorname{tr}M \vert ^{2}}{n} \biggr], \\ &(2)\quad \vert \operatorname{tr}M \vert ^{2}>(n-1) \biggl[ \biggl(\bigl(\varphi_{M}(k)\bigr)^{2}-\frac{1}{2} \bigl\Vert \bigl[M_{k},M_{k}^{*}\bigr] \bigr\Vert ^{2}_{F} \biggr)^{\frac{1}{2}}+\frac{ \vert \operatorname {tr}M \vert ^{2}}{n} \biggr]. \end{aligned}$$

According to Theorem 2.3, we can directly get the following.

### Theorem 3.2

*Let* $M \in M_{n}(C)$; *if* *M* *satisfies one of the following conditions*, *then* *M* *is nonsingular matrix*:

$$\begin{aligned} &(1)\quad \bigl\vert \operatorname{Re}(\operatorname{tr}M) \bigr\vert ^{2}>(n-1) \biggl( \biggl\Vert \frac{M+M^{*}}{2} \biggr\Vert ^{2}_{F}-\frac {1}{2} \Vert M \Vert ^{2}_{F}+\frac{1}{2}\omega(M,x) \biggr), \\ &(2)\quad \bigl\vert \operatorname{Im}(\operatorname{tr}M) \bigr\vert ^{2}>(n-1) \biggl( \biggl\Vert \frac{M+M^{*}}{2\sqrt{-1}} \biggr\Vert ^{2}_{F}-\frac{1}{2} \Vert M \Vert ^{2}_{F}+\frac{1}{2}\omega (M,x) \biggr), \end{aligned}$$

*where* $\omega(M,x)= ((\varphi_{M}(k,x))^{2}-\frac{1}{2} \Vert [M_{k}(x),M_{k}(x)^{*}] \Vert ^{2}_{F} )^{\frac{1}{2}}+\frac { \vert \operatorname{tr}M \vert ^{2}}{n}$.

According to Corollary 2.3, we have the following.

### Corollary 3.2

*Let* $M \in M_{n}(C)$; *if* *M* *satisfies one of the following conditions*, *then* *M* *is nonsingular matrix*:

$$\begin{aligned} &(1)\quad \bigl\vert \operatorname{Re}(\operatorname{tr}M) \bigr\vert ^{2}>(n-1) \biggl( \biggl\Vert \frac{M+M^{*}}{2} \biggr\Vert ^{2}_{F}-\frac {1}{2} \Vert M \Vert ^{2}_{F}+\frac{1}{2}\omega(M,x=1) \biggr), \\ &(2)\quad \bigl\vert \operatorname{Im}(\operatorname{tr}M) \bigr\vert ^{2}>(n-1) \biggl( \biggl\Vert \frac{M-M^{*}}{2\sqrt{-1}} \biggr\Vert ^{2}_{F}-\frac{1}{2} \Vert M \Vert ^{2}_{F}+\frac{1}{2}\omega (M,x=1) \biggr), \\ &(3)\quad \bigl\vert \operatorname{Re}(\operatorname{tr}M) \bigr\vert ^{2}>(n-1) \biggl( \biggl\Vert \frac{M+M^{*}}{2} \biggr\Vert ^{2}_{F}-\frac {1}{2} \Vert M \Vert ^{2}_{F}+\frac{1}{2}\omega(M,x=\sqrt{\mu }) \biggr), \\ &(4)\quad \bigl\vert \operatorname{Im}(\operatorname{tr}M) \bigr\vert ^{2}>(n-1) \biggl( \biggl\Vert \frac{M-M^{*}}{2\sqrt{-1}} \biggr\Vert ^{2}_{F}-\frac{1}{2} \Vert M \Vert ^{2}_{F}+\frac{1}{2}\omega (M,x=\sqrt{\mu}) \biggr), \end{aligned}$$

*where* $\omega(M,x=1)= ( ( \Vert M \Vert ^{2}_{F}-\frac { \vert \operatorname{tr}M \vert ^{2}}{n} )^{2}-\frac {1}{2} \Vert [M,M^{*}] \Vert ^{2}_{F} )^{\frac {1}{2}}+\frac{ \vert \operatorname{tr}M \vert ^{2}}{n}$, $\omega(M,x=\sqrt{\mu})= ((\varphi_{M}(k))^{2}-\frac{1}{2} \Vert [M_{k},M_{k}^{*}] \Vert ^{2}_{F} )^{\frac{1}{2}}+\frac { \vert \operatorname{tr}M \vert ^{2}}{n}$.

Matrix inequality is a research focus in the inequality field and a good many scholars have been researching on this topic. For instance, Hu and Xue [5] obtained some improved reverses of Young type inequalities for matrices, Zou and Peng [6] presented some trace inequalities for matrix means, Zou and Jiang [7] gave a note on interpolation between Cauchy-Schwarz matrix norm inequalities and the arithmetic-geometric mean.

## Conclusion

In matrix analysis, the elements of the matrix to determine the nonsingularity of the matrix have been widely used in practical problems. In this paper, we firstly base our considerations on the Kress theorem in [4], using the partitioned matrices to obtain a new estimation for $\sum_{i=1}^{n} \vert \lambda_{i} \vert ^{2}$. Secondly, through the new estimation mentioned above, some new and more accurate estimations for the lower bound for the rank of the matrix are obtained, such as theorems and corollaries in Section 2. Lastly, due to the nonsingularity of the matrix being closely related to the lower bound for the rank of the matrix, using the results in Section 2, we can get some new sufficient conditions for nonsingular matrices, such as the theorems and corollaries in Section 3.
